# Modeling rare genetic disease with gene-edited induced pluripotent stem cells: relevance of the starting stock line

**DOI:** 10.1093/stcltm/szaf065

**Published:** 2025-12-16

**Authors:** Ashok R Dinasarapu, Diane J Sutcliffe, Erkin Ozel, Anike Thite, Lauren Grychowski, Jasper E Visser, Ellen J Hess, Sharon M Kolk, H A Jinnah

**Affiliations:** Department of Neurology, Emory University School of Medicine, Atlanta, GA 30322, United States; Department of Neurology, Emory University School of Medicine, Atlanta, GA 30322, United States; Department of Neurology, Emory University School of Medicine, Atlanta, GA 30322, United States; Department of Pediatrics, Emory University School of Medicine, Atlanta, GA 30322, United States; Department of Neurology, Emory University School of Medicine, Atlanta, GA 30322, United States; Department of Neurology, Emory University School of Medicine, Atlanta, GA 30322, United States; Neurobiology Section, Donders Center for Neuroscience, Radboud University Nijmegen, Nijmegen, The Netherlands; Department of Neurology, Radboud University Medical Center Nijmegen, Nijmegen, The Netherlands; Department of Neurology, Amphia Hospital, Breda, The Netherlands; Department of Pharmacology, Emory University School of Medicine, Atlanta, GA 30322, United States; Neurobiology Section, Donders Center for Neuroscience, Radboud University Nijmegen, Nijmegen, The Netherlands; Department of Neurology, Emory University School of Medicine, Atlanta, GA 30322, United States; Department of Pediatrics, Emory University School of Medicine, Atlanta, GA 30322, United States; Department of Human Genetics, Emory University School of Medicine, Atlanta, GA 30322, United States

**Keywords:** Lesch–Nyhan disease, HPRT1, hypoxanthine-guanine phosphoribosyltransferase, human induced pluripotent stem cell, disease modeling

## Abstract

Induced pluripotent stem cells (iPSCs) are commonly used to model human genetic diseases. Two main strategies are used. The first involves making iPSC lines from individual cases with a disease, and the second involves making disease-relevant gene edits in established iPSC lines. Because generating gene-edited lines is time consuming and expensive, most studies begin with one starting iPSC stock line and evaluate several gene-edited sublines. The current studies focus on gene-editing to model Lesch–Nyhan disease (LND), which is caused by mutations in the *HPRT1* gene. The same pathogenic c.508C>T edit was made in four well-established stock lines, and three gene-edited lines were isolated from each. RNA sequencing (RNAseq) was, then, used to evaluate the impact of the gene edit. Gene-edited lines were compared to their corresponding stock lines, as well as to each other. An aggregate analysis of all lines combined was also conducted to determine the most robust findings across all lines. Results from gene editing were further compared with iPSC lines derived from individual cases with LND, to determine how closely findings from gene editing match results obtained with case-derived lines. There were two main findings. First, the same gene edit has a different impact on gene expression when starting with different starting stock lines. Second, the gene editing strategy does not produce the same results as the case-derived strategy. Potential explanations for these differences are addressed, along with the relevance of these two different strategies for disease modeling.

## Graphical abstract

**Graphical Abstract:**
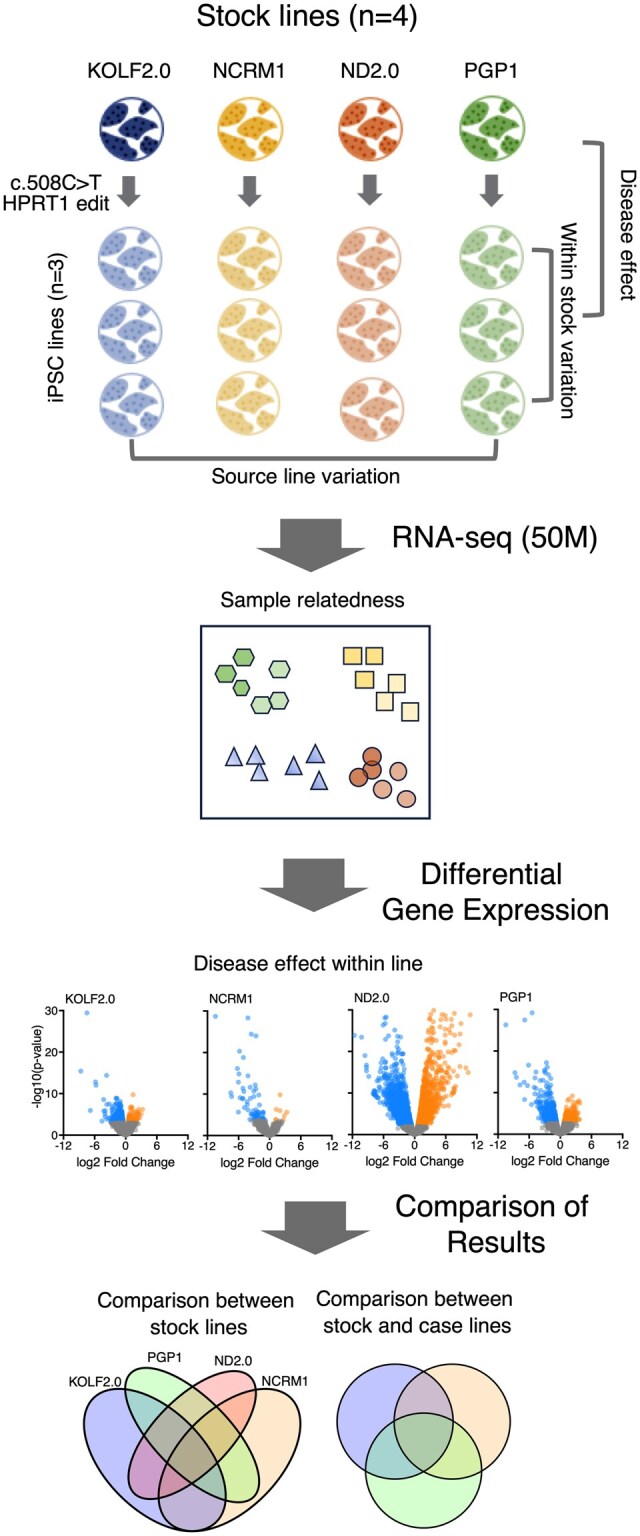
Modeling of Lesch–Nyhan disease was conducted by starting with four different stock iPSC lines and preparing three sublines from each with the same gene edit. The proper c.508C>T gene edit producing a premature stop codon in the *HPRT1* gene and its effect on enzyme activity was confirmed for all edited lines. Next, all gene edited lines were compared to their corresponding stock lines, as well as each other. Results from each line were also compared with results from an overall analysis of all lines combined, to mitigate idiosyncratic differences among individual stock lines. Results from each gene edited stock line and all gene edited lines combined were also compared with a previously reported dataset involving a large bank of iPSC lines derived directly from individuals with the disease. Finally, sources of variance were explored.

Significance Statement Stem cells provide valuable tools for studying genetic diseases. However, they can be challenging to make from people with diseases, especially when the diseases are rare. An alternative strategy is to genetically engineer stem cells to match a genetic disease. The current study uses genetic engineering to make stem cells for Lesch–Nyhan disease. It compares results obtained from engineering stem cells from different sources and compares results to what is found for stem cells from patients.

## Introduction

Induced pluripotent stem cells (iPSCs) have become very popular for modeling human diseases.^1–3^ For genetic diseases, two main strategies are used. The first involves collecting somatic cells from individuals with a disease and converting them into pluripotent cells (case-derived iPSCs). The second involves making disease-relevant gene edits in iPSC lines prepared from healthy controls (gene-edited iPSCs). The case-derived and gene-editing strategies for disease modeling have different strengths and weaknesses.

An obvious strength of the case-derived strategy is that iPSCs are produced from an individual with a phenotype known to be caused by a specific pathogenic gene variant. A disadvantage of the case-derived strategy is that it is often difficult to find enough affected individuals, especially when the disease is rare. Another disadvantage is experimental variability among lines prepared from different individuals,^4–9^ leading to uncertainty regarding how many independent lines need to be studied.^10–14^ A third disadvantage is that preparing case-derived lines is labor-intensive and expensive. As a result, prior studies using the case-derived iPSC strategy have often included only 1-3 cases per group, with most including only 1-2 sublines per case.^11^^,^^14^

By comparison, the gene-editing strategy most often involves starting with iPSCs from a well-established stock line initially made from a healthy individual and then introducing a genetic change that is disease-relevant. It is also feasible to start with iPSCs from individuals with a disease and correct the existing genetic defect (gene-corrected iPSCs), although this strategy is less common. The main strength of these gene editing strategies is that variability inherent in comparing lines from different individuals is reduced, because the effects of the gene edit can be compared with the starting line to mitigate the impact of different genetic backgrounds or different reprogramming efficiencies.^10^^,^^15^^,^^16^ Some disadvantages of gene-edited lines are that the editing process may introduce off-target genetic changes, stock lines have adaptations for long-term growth in culture which may impact the disease phenotype, and the gene editing process requires isolation of individual sublines that may differ from the parent culture in ways unrelated to the gene edit.

For case-derived lines, several reports have suggested the most robust outcomes require inclusion of at least four unrelated individuals per group, and avoiding the use of multiple iPSC lines from the same case.^14^^,^^16–19^ Similar guidance regarding the optimal numbers of gene-edited sublines and starting stock lines is lacking. The most common strategy has been to study 1-3 gene-edited sublines derived from one stock line.^20^ Empirical data regarding the consequences of using only one starting stock line are lacking. Also lacking are methodical comparisons of results obtained using the case-derived or gene-editing strategies.

To address some of these knowledge gaps regarding gene-editing strategy for disease modeling, the same pathogenic gene edit in the *HPRT1* gene (c.508C > T producing a premature translational stop codon) associated with Lesch–Nyhan disease (LND) was introduced into four different publicly available stock iPSC lines. Three independent gene-edited sublines were isolated for each of the four stock lines. RNA sequencing (RNAseq) was then used to evaluate the impact of the gene edits. The effect of the gene edit was compared across the different stock lines, as well as with a large group of previously described case-derived lines.

## Methods

### Generation of gene-edited iPSC lines

The overall study design is outlined in the Graphical Abstract. Four widely used parental stock lines were selected from public biobanks (Table 1). All were from male individuals, so that a single allele could be targeted for the X-linked *HPRT1* gene. The initially selected stock lines included KOLF2.0, NCRM-1, NN5200, and PGP1. Following gene editing, virtually all NN5200 sublines were found to have a 20q+ in the Karyoscan. As a result, NN5200 was replaced with an alternative male iPSC line, ND2.0.

**Table 1.** iPSC stock lines used for editing.

Stock line name	Source ID	Sex	Age	Sample source	Reprogram method	*HPRT1* gene	HGprt enzyme	Sample citations	Subsample	*HPRT1* qPCR
**KOLF2.0**	HPSIO114i-kolf_2	male	55 yrs	fibroblast	Sendai virus	normal	23.1	^20–24^	A	42.8
									B	53.6
									C	59.3
**NCRM1**	GM23338	male	1 day	cord blood	episome	normal	56.4	^25–29^	A	46.5
									B	54.6
									C	55.7
**ND2.0**	ND50028	male	NA	fibroblast	episome	normal	35.4	^9^ ^,^ ^30–36^	A	27.8
									B	55.2
									C	40.3
**PGP1**	ND50019	male	55 yrs	fibroblast	retrovirus	normal	45.4	^37–43^	A	32.1
									B	51.5
									C	62.3

Age refers to subject age at source sample collection. *HPRT1* mRNA levels are relative values from qPCR. Enzyme activity was measured as previously described.^44^

A pathogenic c.508C > T premature translational termination codon in *HPRT1* (p.Arg170Ter) associated with the typical LND clinical phenotype in multiple unrelated cases^45^ was introduced into each stock line. The same genetic variant was introduced for all lines because the goal was to assess variability related to standard gene-editing methods rather than variability associated with different *HPRT1* mutations. This mutation predictably reduces the mRNA transcript, because it is subject to nonsense-mediated decay. The mutation also predictably eliminates the activity of the associated enzyme (hypoxanthine-guanine phosphoribosyltransferase, HGprt), which has known function in purine metabolism.^46^^,^^47^ A total of three independent gene-edited sublines were generated for each of the four starting stock lines (total *N* = 12 gene-edited lines).

For gene editing, two different editing methods were used to address the possibility that the editing method itself might be the main variable responsible for any findings. Two stock lines (PGP1 and NCRM-1) were prepared using homologous direct repair paired with SNIPER technology (Reprocell; https://www.reprocell.com/blog/improved-gene-editing-with-crispr-sniper). Briefly, cells in multi-well plates were electroporated with a Cas9 plasmid, single-guide RNA expression plasmid (*HPRT1*-AS16705: 5′-GGCTTATCCAACACTTCG-3′), and donor plasmid. Cells from different wells were screened by electrophoresis to detect the N1aIII-sensitive c.508C>T edit. Genomic DNA from screened samples was used as a template for PCR confirmation of the target sequence. Final selected gene-edited clones were karyotyped at 400 band resolution (clgenetics.com), and the intended edit was confirmed by Sanger sequencing of PCR-amplified genomic DNA.

The other two stock lines (ND2.0 and KOLF2.0) were prepared by electroporating different synthetic guide RNAs along with a single-stranded oligodeoxynucleotide donor with Cas9 (Synthego; www.synthego.com). Editing efficiency was assessed 48 h after electroporation. Genomic DNA was extracted, amplified by PCR, and Sanger sequenced. The resulting chromatograms were processed using Synthego Inference of CRISPR edits software (ice.synthego.com). To generate monoclonal lines, cells were seeded at 1 cell/well into 96 or 384 well plates and imaged every 3 days to ensure expansion of a single clone. To ensure lines came from independent editing events, only one gene-edited line was selected per edited pool. Clonal populations were screened and identified using the PCR-Sanger-ICE genotyping strategy and Sanger sequencing to verify the correct mutation. Chromosomal integrity was assessed using the KaryoStat array (www.thermo-fisher.com/karyostat).

In addition to verifying the intended mutations by Sanger sequencing, each gene-edited line was subject to further checks after expansion (Table 2). In keeping with current recommendations,^48^ these checks included re-assessment of the karyotype and confirmation of intended mutation. The RNAseq data were also interrogated for gene expression patterns consistent with typical pluripotent cell lines.

**Table 2.** *HPRT1* gene-edited iPSC lines.

Stock line	Subline ID	Pluripotency genes expressed in RNAseq	Genome integrity	*HPRT1* qPCR	qPCR *HPRT1* c508C > T edit	RNAseq *HPRT1* c.508C > T edit	HGprt enzyme
**KOLF2.0**	A12	yes	Karyostat	8.3	confirmed	confirmed	ND
	A14	yes	Karyostat	7.0	confirmed	confirmed	ND
	J11	yes	Karyostat	7.7	confirmed	confirmed	ND
**NCRM1**	E4B2	yes	Karyotype	8.8	confirmed	confirmed	ND
	C9E4	yes	Karyotype	10.0	confirmed	confirmed	ND
	B1	yes	Karyotype	6.4	confirmed	confirmed	ND
**ND2.0**	B7	yes	Karyostat	4.4	confirmed	confirmed	ND
	C5	yes	Karyostat	4.0	confirmed	confirmed	ND
	H4	yes	Karyostat	4.4	confirmed	confirmed	ND
**PGP1**	H3	yes	Karyotype	10.0	confirmed	confirmed	ND
	C7H1	yes	Karyotype	5.0	confirmed	confirmed	ND
	B3	yes	Karyotype	9.6	confirmed	confirmed	ND

*HPRT1* mRNA levels are relative values from qPCR. Enzyme activity was measured as previously described (ND = not detectable).^44^

### Bulk gene expression profiling

All cultures were grown and harvested by the same technician using the same methods and the same standard tissue culture medium (mTeSR1 medium, Stemcell Technologies). Bulk RNAseq was conducted for all samples together in a single batch.^44^ The sequencing target was 50 million paired-end, 100 bp reads for each sample. Final reads fell between 50 and 75 million (average 60 million). The quality of reads was verified by FastQC v0.11.4 and aligned with the human reference genome (UCSC RefGene; hg38 build with 26 485 genes) using STAR (Spliced Transcripts Alignment to a Reference) read mapper.^49^ Overall, 92%-94% of uniquely mapped reads counted using STAR option *quantMode GeneCounts*. Downstream analyses involved R Statistical Software (v4.2.3; R Foundation for Statistical Computing, Vienna, Austria) in RStudio (v2023.03.1 Build 446). Normalization of read counts was done using the trimmed mean of *M*-values (TMM) with the edgeR v3.40.2 R package^50^ and converted to log_2_ counts per million (CPM).

### Analytical strategy for differential gene expression

For bulk gene expression, principal component analysis (PCA) was initially done using normalized and log2 transformed CPM, with both centering and scaling enabled to identify relatedness among different iPSC lines using the prcomp method from the stats v4.2.3 R package. Results were visualized with the R package ggplot2 v3.4.2. Variance partitioning with the matrix of normalized gene counts via fitExtractVarPartModel and plotVarPart from the R package variancePartition v1.32.5 was used to identify major sources of variance in gene expression.^51^

Multiple methods are available to detect differentially expressed genes. Among these, two were selected, edgeR and limma-voom. The purpose was not to compare and contrast lists of genes revealed by these two methods, but to determine if they produced different overall conclusions. These two methods are based on different statistical models, with distinct methods for handling genes with low counts, distinct methods for normalization, and distinct methods for estimating variance. EdgeR relies on a negative binomial distribution developed for RNAseq data^11^^,^^50^ Implementation involved the likelihood ratio test using glmFit and glmLRT. Post hoc pairwise tests were done using exactTest.^50^ EdgeR was also used with a design matrix that accounted for baseline differences among stock lines to detect gene editing effects independent of cell line. The limma method was originally developed for microarray data, and later adapted for RNAseq as limma-voom. Analyses were conducted using limma-voom with the v3.60.6 R package,^52^ before and after correction for replicate sublines from the same stock line using duplicateCorrelation (DupCor) with a block factor adjusted for cell lines which isolate editing effects from cell line variations. For both edgeR and limma-voom, the *P*-value was adjusted according to the false discovery rate (FDR) with FDR < 0.05 considered significant.^53^ Results are shown as volcano plots generated with GraphPad Prism v9.5.1.

To obtain a fuller understanding of the relationships among changes in expression of individual genes, the Gene Set Enrichment Analysis (GSEA) was performed using v4.3.3 (build 16) stand-alone tool with Gene Ontology (Biological Process) annotations (c5.go.bp.v2014.1.Hs.symbols.gmt), ranking genes by the Signal2Noise metric.

### Performance metrics for sensitivity and specificity

To determine the performance of different analytical methods for detecting differential gene expression, the stock and gene-edited samples were shuffled systematically under the null hypothesis that they all come from the same distribution. Fifty genes were selected at random and given pre-defined fold changes (1.5X, 2X, 3X, 5X, and their inverses) for a total of 400 changes. Differential gene expression analyses were done following systematic permutations of all possible combinations of samples. Based on the known changes, true positives and false positives were compared using different analytical methods and different combinations of iPSC sublines.^11^ The optimal balance between sensitivity and specificity was assessed using the area under the curve metric (AUC).^11^

### Single-cell RNA sequencing

For single-cell RNA sequencing (scRNAseq), iPSC colonies were dissociated using Accutase (StemPro™) and seeded at 100 000 cells/mL on Matrigel coated 6-well dishes as previously described.^44^ At 90%-100% confluency, cells were incubated in 1 mL Accutase per well for 20 min at 37 °C and removed from 6-well culture plates with a 5 mL pipette. Cells were then transferred into 10 mL sterile centrifuge tubes and gently pipetted up and down for 10 times to obtain single cells. Next, cells were centrifuged at 500 rcf for 3 min to pellet cells. Supernatant was removed and cells were washed with 0.04% BSA in PBS 3 times. Cells in 0.04% BSA-PBS were filtered through a 40 µm cell strainer (#2236347 Fisherbrand™ Sterile Cell Strainer) to remove cell clumps and debris. Finally, cells were counted and assessed for viability and dissociation using an automated cell counter (Invitrogen Countess 3 Automated Cell Counter) with trypan blue exclusion. The resulting single-cell suspension was aliquoted in 500 µL 0.04% BSA-PBS with a target cell concentration of 7 × 10^5^ cell/mL and viability above 90%. Cells were kept on ice for a maximum of 30 min until proceeding with library preparation. Gene expression profiles were obtained using 10X Genomics Chromium Single Cell technologies. NovaSeq S1 Flow Cell Sequencing System was used to yield 80 000 reads/cell with a target cell count of 10 000.

The raw scRNAseq reads in FASTQ were processed using CellRanger v7.2.0 (10X Genomics) and aligned to the Human reference (GRCh38)-2020-A. The resulting raw gene-cell Unique Molecular Identifiers (UMI) matrix from each sample was converted to corresponding Seurat object using Read10X function from Seurat v4.40 R package.^54^ To avoid confounding effects due to low quality cells, cells were excluded with >9% of total UMIs (nCount_RNA) from mitochondrial genes and <500 features (nFeature_RNA) before proceeding with normalization. The differentially expressed genes between cell subpopulations were discovered utilizing the FindMarkers function with parameters logfc.threshold = 0.25 and only.pos = TRUE in Seurat, employing the default Wilcoxon rank sum test. The gene list was subsequently filtered by the following criteria: gene was detected in >85% cells from the first group (pct.1 > 0.85) and adjusted *P*-value < .01. Gene Ontology enrichment analysis was performed for the identified differentially expressed genes using clusterProfiler R package. A heatmap was created for genes with average log_2_ fold change > 2.0.

## Results

### Gene-edited lines have expected consequences for *HPRT1*

**Figure 1.**
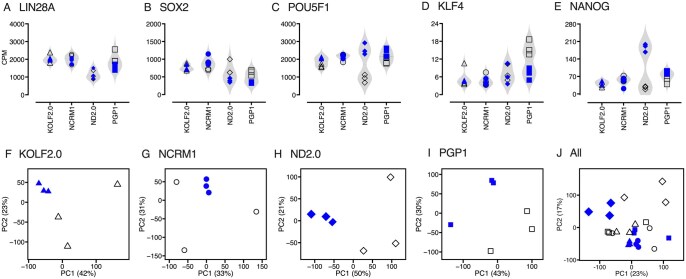
Gene expression across all stock and gene-edited iPSC lines. (A–E) depict expression of typical pluripotency genes for each line as violin plots. (F–I) show relatedness of individual stock lines and their derivatives and (J) shows relatedness of all samples as a principal component analysis plot with only first two components PC1 and PC2 shown. The unedited stock samples are each shown as a different type of open symbol, and their gene-edited counterparts are shown as closed symbols (triangle = KOLF2.0, circle = NCRM1, square = PGP1, diamond = ND2.0).

The same pathogenic c.508C > T mutation causing a premature stop codon in the *HPRT1* gene was introduced into four widely used stock iPSC lines (Table 1), with three independent gene-edited lines isolated for each (*N* = 12 gene-edited lines). Three independently cultured samples of each of the four stock lines were used as controls (*N* = 12 control samples). Interrogation of RNAseq data revealed detectable expression of expected pluripotency genes across all samples (Figure 1A–E). There were no consistent differences in expression of these genes across the different stock lines or following gene editing. However, one stock line (PGP1) showed apparently lower levels of one gene (*KLF4*) after gene-editing and another line (ND2.0) showed apparently higher levels of two other genes (*POU5F1* and *NANOG*) after gene editing (Figure 1C–E). These changes may reflect the isolation of gene-edited subclones, a process that may select for growth habits that are different from the broader parent population (as described further below for the ND2.0 line).

The RNAseq data were also used to confirm the intended c.508C > T mutation in the *HPRT1* sequence (Table 2 and Supplementary Figure S1). The impact of the genomic mutation was also confirmed by RT-PCR, which further demonstrated low *HPRT1* mRNA levels, as expected for a stop codon that leads to accelerated nonsense-mediated decay (Tables 1 and 2). The enzymatic activity of HGprt was confirmed to be positive for all starting stock lines and negative for all gene-edited lines using a radiometric enzyme assay (Tables 1 and 2).^46^

### The same gene edit produces different results in different starting stock lines

The most common gene-editing strategy is to generate 1-3 separate gene-edited lines, usually all from a single stock line.^20^ Statistical comparisons are then most often based on comparing technical replicates of the gene-edited lines with technical replicates of the starting line. The assumption is that the same gene edit in different stock lines should have similar effects on gene expression. To test this assumption, RNAseq results were normalized for each stock line and its corresponding gene-edited line, and PCA was used to show sample relatedness. The PCA plots imply that gene editing had a significant impact on the transcriptome of each line (Figure 1F–I). However, separating the stock lines into different PCA plots makes it difficult to know if these gene expression changes were similar across the stock lines because individual genes contributing variance are not the same in each plot. When all results are combined into a single plot to address this limitation, the gene edited lines do not appear to show clear segregation from their corresponding stock lines (Figure 1J). Instead, a unique gene expression pattern emerges for the ND2.0 line, with or without gene edits, as shown by its segregation from the other lines on PC2 (Figure 1J).

**Figure 2.**
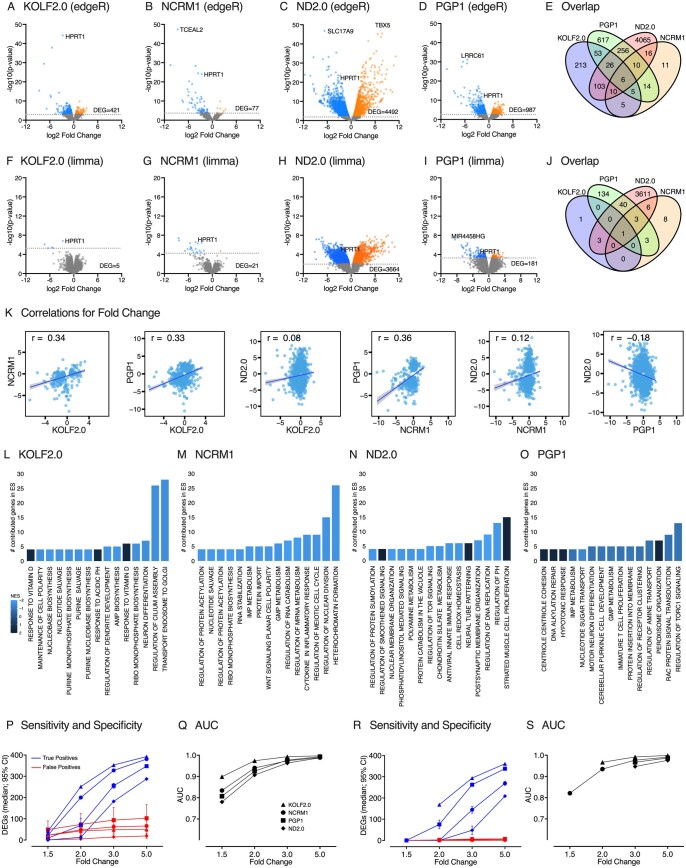
Differential gene expression for each gene-edited iPSC stock line. (A–D) show differential gene expression as volcano plots when applying edgeR to the three gene-edited lines as compared to three replicates of the corresponding normal stock line. (E) shows the overlap in statistically significant genes at FDR < 0.05 across the four gene-edited groups. (F–J) show similar analyses when applying limma-voom instead of edgeR. (K) shows Pearson correlations among the four stock lines for the fold-change in genes differentially expressed in any stock line. (L–O) show biological pathways impacted by gene editing in each of the four stock lines, as defined by the gene set enrichment method. (P–S) show performance metrics when known changes in gene expression are introduced into the dataset for each of the stock lines when applying edgeR (P, O) or limma-voom (R, S) in terms of sensitivity, specificity, and the area under the curve (AUC) showing the optimal balance between sensitivity and specificity.

Next, edgeR was used to detect differences in expression of individual genes within each of the four groups. Using this approach, the same gene edit produced significant differences in expression of certain genes at FDR < 0.05 for each line: KOLF2.0 = 421, NCRM1 = 77, ND2.0 = 4492, and PGP1 = 987 (Figure 2A–D). There was surprisingly little overlap among the genes detected across the different stock lines (Figure 2E) with only six genes in common. Among these six genes, *HPRT1* was reduced across all four gene-edited stock lines, as expected for a stop codon that is subject to nonsense-mediated decay, and confirmed by independent RT-PCR (Tables 1 and 2). Also consistently reduced across all lines were *KCNA1* and *PFKFB3*. Two genes (*RBM46* and *LINC01139*) had a consistent direction of change in three lines, but went in the opposite direction for the ND2.0 line. Therefore, only four genes showed consistent effects of gene editing across all four stock lines.

The limma-voom method, which is based on very different statistical assumptions, was also applied to determine if it might provide a more consistent result for gene editing across the four stock lines. Compared to edgeR, fewer genes were differentially expressed using limma-voom: KOLF2.0 = 5, NCRM1 = 21, ND2.0 = 3664, PGP1 = 181 (Figure 2F–I). There was again limited overlap among genes found for different stock lines (Figure 2J) with only one gene shared among all lines (*HPRT1*). These results confirm prior studies that the limma-voom method is more conservative than edgeR,^19^ but there was still limited overlap when the same gene edit is made in four different stock lines. Similar differences were found among stock lines when all gene-edited (*N* = 12) and stock samples (*N* = 12) were normalized in a single batch (instead of separately) and followed by contrasts performed for each stock line to assess editing effects (not shown).

The unexpectedly low overlap in gene expression changes across the four gene-edited stock lines may reflect in part the arbitrary statistical cutoffs used to define statistical significance. For example, a large number of genes fell in the range of 0.05 < FDR < 0.10 for using either edgeR or limma-voom. To explore this potential explanation for limited overlap, changes in the expression of all genes across all gene-edited lines were evaluated using Pearson correlations, regardless of whether the genes met the statistical criteria of FDR < 0.05 for differential expression. There were statistically significant correlations among all lines (Figure 2K). However, the strength of the correlations was moderate for three lines (KOLF2.0, NCRM1, PGP1) at *r *= 0.33 to 0.36, suggesting only moderate similarity of gene editing across these lines. Correlations between the ND2.0 and other lines were weak (*r* = 0.08 to −0.18), suggesting limited similarity for ND2.0. Although the *P*-values for all correlations were highly significant due to large sample sizes, the correlation coefficients indicate only moderate biological similarity of gene edits, and only for KOLF2.0, NCRM1, and PGP1.

Another explanation for the limited overlap is that the biological pathways are affected by the gene edit are similar across different stock lines, even when individual genes within those pathways do not consistently reach the predefined criteria for statistical significance. To address this possibility, the GSEA method was used for biological pathway analyses as it considers ranked gene lists rather than relying on arbitrary statistical cutoffs used to define a differentially expressed genes. Among the top 15 biological pathways identified for each stock line, there was limited overlap among the four stock lines, although purine-related pathways were identified for three of four lines (Figure 2L–O).

Overall, these findings raise questions regarding assumptions that the same gene edit should produce similar results in different stock lines, because of substantial variation in the lists of genes differentially expressed across the four stock lines, the modest correlations for changes in gene expression across these lines, and limited overlap in biological pathways impacted by the gene edits.

### Performance metrics for detecting gene expression changes among different stock lines

One limitation of the analyses above is that actual changes for gene expression expected of the gene edits are unknown, except for *HPRT1*. For other genes, it is impossible to know which stock line is producing the most accurate result, and many of the differentially expressed genes may reflect false positives (noise). To evaluate this possibility, all gene edited lines and their corresponding stock samples were systematically shuffled under the null hypothesis that they all have the same random distributions of gene expression.^11^ For each of the four stock line groups, pre-defined changes (fold changes of 1.5, 2, 3, 5, and their inverses) were made for 50 randomly selected genes, totaling 400 gene changes. Differential gene expression analyses were then conducted to find these changes, employing systematic permutations of all possible combinations of the six samples within each of the four stock line groups.

False positives did not change with fold-change introduced, because they depend more on the random groupings of samples during permutations. False positives were higher when using edgeR (Figure 2P and Q) compared to limma-voom (Figure 2R and S), confirming the latter to be statistically more conservative. However, sensitivity for detecting true positives was low for both analytical methods, most likely because statistical power was limited by the small numbers of samples for comparisons (*N* = 3 stock control samples and *N* = 3 gene edited samples). Sensitivity was lower for the limma-voom method; fewer than half of the introduced changes could be detected in any stock line even with a fold change of 2.0. More importantly, the sensitivity for detecting true positives differed substantially across the stock lines, most likely because of differences in the degree of variance in gene expression inherent within each stock line. The highest numbers of true positives were detected with KOLF2.0 and the lowest with ND2.0 (Figure 2P–S). These results indicate that the likelihood of false positive findings depends on the starting stock line used and the fold-change observed. For some lines (PGP1, ND2.0) false positives were more common than true positives at fold-changes of 1.5 or 2.0.

### Aggregate analysis across all iPSC lines as “common standard”

**Figure 3.**
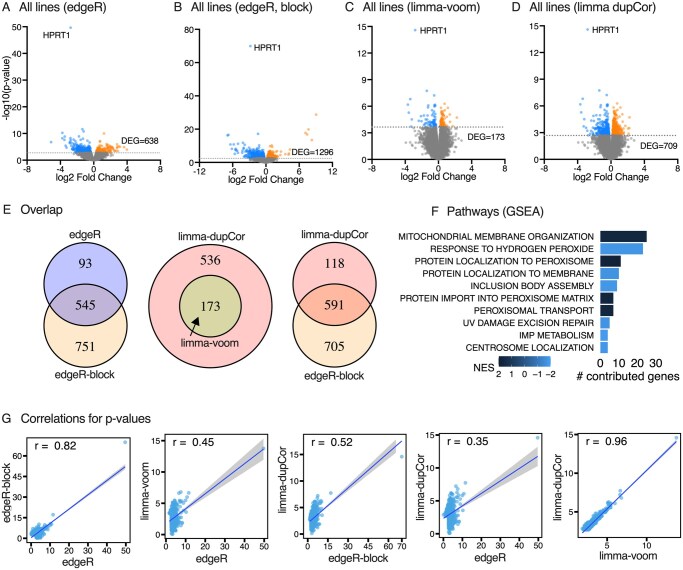
Differential gene expression after aggregating results across all gene-edited stock lines. (A–D) show differential gene expression as volcano plots when applying two different analytical methods: edgeR (with and without adjusting for stock line variance) or limma-voom (with and without DupCor to account for stock line variance). (E) is a Venn diagram depicting the overlap of results for these different methods. (F) shows the biological pathways defined by Gene Set Enrichment Analysis (GSEA) which evaluates the entire gene list by rank and does not rely on differentially expressed genes only. (G) shows correlations for *P*-values across the different methods. For the second correlation plot, HPRT1 falls withing the confidence interval, and removal of HPRT1 drops the correlation coefficient to 0.25. In the other plots, HPRT1 does not fall within the confidence interval, and removing it has no effect.

Studies of disease modeling using gene-edited iPSC lines do not often evaluate multiple different starting lines. Under the assumption that an overall aggregate analysis of all lines would identify the most consistent and robust changes in gene expression caused by the c.508C>T edit (regardless of the starting stock line), all stock samples (*N* = 12) and all gene-edited lines (*N* = 12) were next normalized together and combined in a single analysis. With edgeR applied to all samples as if they were independent, there were significant differences for 638 genes (Figure 3A). When evaluated with a design matrix that included both cell line and editing status as separate factors so that baseline stock cell differences were taken into account, edgeR detected significant differences for 1296 genes (Figure 3B). With or without correction for stock line, the top hit was *HPRT1,* as expected (Figure 3A and B).

Limma-voom without correction for stock line revealed significant differences for 173 genes across the gene-edited group compared with unedited stocks (Figure 3C). Application of DupCor with a block factor adjusted for stock lines to isolate editing effects from stock line variations increased the number of differentially expressed genes to 709 (Figure 3D). Independent of stock line blocking, *HPRT1* was again the top hit (Figure 3C and D).

Results from applying edgeR or limma-voom were also compared with each other, with and without correction for stock line relatedness (Figure 3E). The majority of genes detected overlapped when comparing edgeR with and without a block design, and with limma-voom with and without DupCor. However, the majority of genes found using the edgeR block design did not overlap with those found using limma-voom with DupCor. These findings indicate that these methods can produce very different results. For these aggregate analyses of all edited stock lines combined, the main biological pathways identified by GSEA were not clearly related to known functions of HGprt in purine metabolism, except for “IMP metabolic process” (Figure 3F).

Pearson correlations for fold changes for individual genes across these methods could not be conducted, since the source data were the same. However, Pearson correlations for *P*-values showed strong positive correlations when results from edgeR with or without block design were compared, and excellent correlations when results from limma-voom with or without DupCor were compared (Figure 3G). Correlations across the edgeR and limma-voom methods were only moderate.

The analyses of all gene-edited lines combined along with statistical correction for relatedness among stock lines was, then, used as a common reference standard to estimate sensitivity and specificity of findings for each stock line separately. The aggregate edgeR analysis of all stock lines and edited counterparts with block matrix design accounting for variations among starting stock lines revealed 1296 total genes. When considering edgeR analyses applied to each stock line separately, the detection of true positives from this list ranged from 2% to 32% (Table 3). For each line, false positives were always greater than true positives, ranging from 2% to 91%. The aggregate limma-voom analysis of all gene-edited lines combined with DupCor yielded 709 genes. The detection of true positives from this list ranged from 0.3% to 44% for individual limma-voom analyses for each stock line separately (Table 3). False positives were again always greater than true positives and ranged from 60% to 92%. If the aggregate analyses are considered a “gold standard,” these results imply that false positives are greater than true positives if only a single stock line is chosen to study differential gene expression with three edited sublines. These results highlight the limitations of using a single starting stock line and evaluating only small numbers of gene edited sublines.

**Table 3.** Performance of individual stock lines against stock-line adjusted aggregate results.

Stock line	EdgeR (total *N* = 1296)			Limma-voom (total *N *= 709)		
	Total genes significant	True positives	False positives	Total genes significant	True positives	False positives
**KOLF2.0**	421	105 (8.1%)	316 (75.0%)	5	2 (0.3%)	3 (60.0%)
**NCRM1**	77	31 (2.4%)	46 (1.5%)	21	8 (1.1%)	13 (62.0%)
**ND2.0**	4492	420 (32.4%)	4072 (90.6%)	3664	312 (44.0%)	3352 (91.5%)
**PGP1**	987	134 (10.3%)	853 (86.4%)	181	28 (4.0%)	153 (84.5%)

### Comparison with a bank of case-derived lines as “reference standard”

In principle, results from gene-edited iPSCs should provide results similar to those obtained when using iPSC lines derived from people with the same genetic disease. Results for differential gene expression obtained from the gene-edited lines were, therefore, compared with results obtained in an independent study comparing iPSCs derived from LND cases versus healthy controls, using the same RNAseq library size and analytical methods.^19^

**Figure 4.**
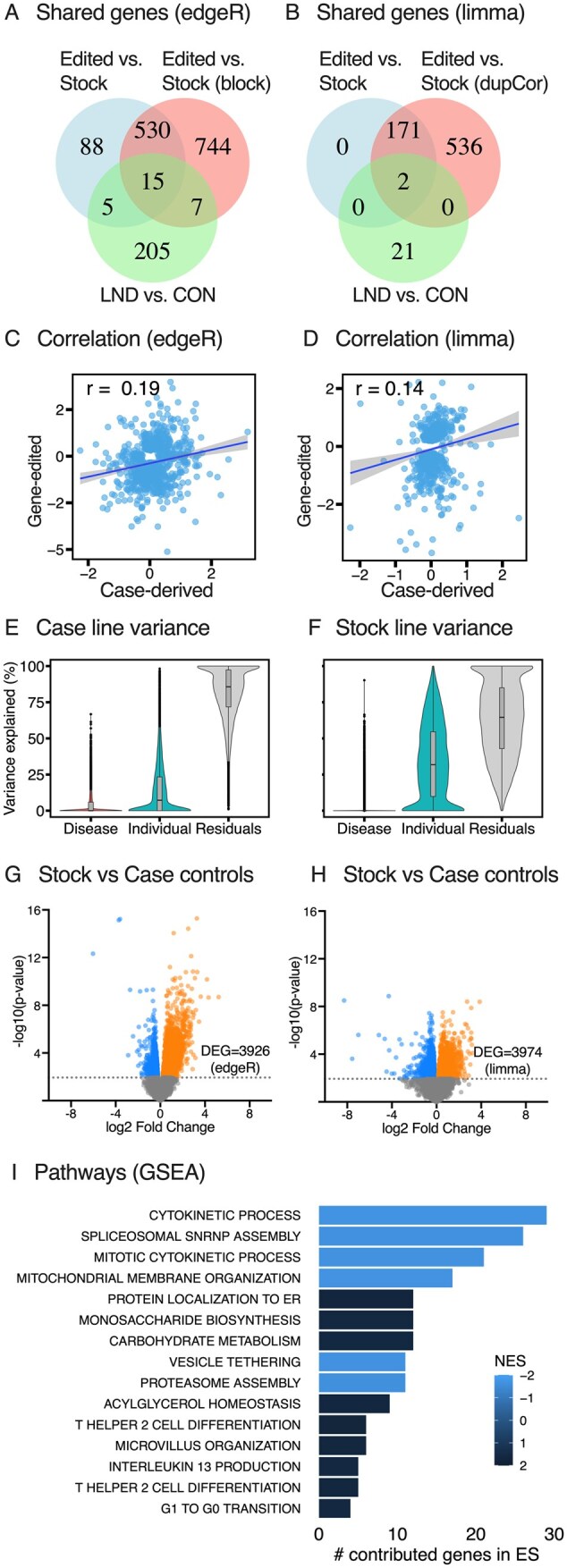
Comparison of case-derived and gene-edited strategies. There was limited overlap among differentially expressed genes (DEG) when the gene-editing and case-derived disease modeling strategies were compared, using either edgeR (A) or limma-voom (B) as the analytical methods when considering all samples to be independent. Overlap did not improve after adjusting for stock line variability (not shown). Correlations for changes in gene expression in the gene-edited and case-derived lines were modest with either edgeR (C) or limma-voom (D) when samples were considered independent, and did not improve substantially after accounting for sample relatedness. (E–F) show the main sources of variance for the case-derived vs gene-editing strategies when using Variance Partitioning. The source lines used to define the baseline for “normal” were significantly different for the two strategies, with 3926 genes differing in edgeR analysis (G), and 3974 genes differing in limma-voom analysis (H). The main biological pathways defined by Gene Set Enrichment Analysis (GSEA) discriminating the normal stock lines from the normal case-derived lines involved DNA replication and cell cycle (I). Abbreviations: ES, enrichment score; NES, normalized enrichment score.

When comparing 12 LND versus 12 control case-derived lines as independent samples, significant differences were found for 232 genes using edgeR and 23 genes using limma-voom.^19^ There was surprisingly little overlap in the lists of significantly affected genes between results obtained from the gene-editing strategy versus the case-derived strategy for disease modeling, no matter which analytical method was applied (Figure 4A and B). Only 15 genes overlapped with edgeR (*HPRT1, SLC2A1, PIWIL2, RNASEL, PCDHBI8P, THAP9-AS1, DNAJA1, VWC2, AURKAPS1, ZNF681, GCNT4, PAX5, ATXN7L2, NR4A2, NUPR1, CARD9, MOB3B*) and only two (*HPRT1, MOB3B*) overlapped with limma-voom.

Three factors contributed to different results for the gene-edited and case-derived iPSC sample sets. The first is related to the arbitrary statistical criteria typically used to define differentially expressed genes. There were significant but modest correlations for the fold-change in expression across all genes found using the gene-edited and case-derived strategies (Figure 4C and D). Therefore, the effect of the disease on overall changes in gene expression between the gene-editing and case-derived strategies was moderately similar, even when differences for individual genes did not reach statistical criteria for significance.

The second factor contributing to discrepancies between the gene-edited and case-derived strategies relates to the main sources of variance in gene expression. For both strategies, variance partitioning revealed that the disease effect contributed only minor variance (Figure 4E and F), implying that *HPRT1* mutations do not have a strong impact on global gene expression. Variance was considerable among individual cases for the case-derived lines. However, variance among stock lines was greater, most likely because they combine differences in starting genetic background, combined with added technical differences relating to reprogramming and long-term maintenance in vitro. However, the largest variance from both the case-derived and gene-editing strategies came from residual (unknown) factors (Figure 4E and F).

The third factor explaining the different results between the gene-edited and case-derived strategies is that two different types of control lines used to define the baseline of “normal” from which to compare the effect of *HPRT1* mutations differed substantially in gene expression. When comparing all unedited (normal) stock iPSC line samples with all case-derived healthy control (normal) iPSC lines, there were very large differences in gene expression in analyses that considered all samples as independent biological replicates (edgeR = 3926; limma-voom = 3974; Figure 4G and H). GSEA revealed the main differences between these two types of “normal” controls involved biological pathways for cell growth (DNA replication and cell cycle), a finding that is not surprising considering that the stock lines that become most popular reflect their robust growth in culture (Figure 4I). These findings imply substantial differences in the starting point for detecting effects of gene editing when using stock iPSC lines versus healthy control iPSC lines.

### Exploration of atypical results from ND2.0 lines

**Figure 5.**
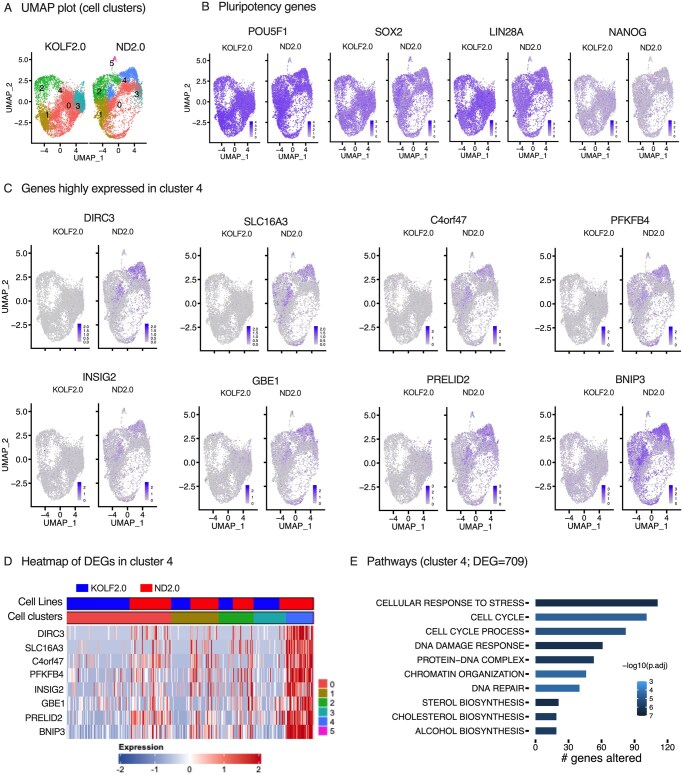
Single-cell RNAseq comparing KOLF2.0 and ND2.0. (A) shows a representative uniform manifold approximation and projection (UMAP) plot with six distinct clusters, with cluster 4 (blue) appearing in ND2.0 but not in KOLF2.0. (B) confirms both lines express typical pluripotency genes (purple). (C, D) show feature plots and a heatmap of genes differentially expressed (DEG) in cluster 4 compared to all other clusters (with fold change >2). (E) shows the biological pathways defining cluster 4 in relation to other clusters. Feature (gene) plots overlay gene expression for each cell on UMAP using a continuous color gradient from light (0, low) to intense (4, high).

Results from the ND2.0 line were subject to further scrutiny for several reasons. The PCA plot indicated this line was different from the other stock lines with or without gene edits (Figure 1J), gene-editing in ND2.0 produced a very large number of differentially expressed genes that did not correlate well with results from other lines (Figure 2K), ND2.0 showed the lowest performance metrics for detecting changes (Figure 2P–S), and two significantly altered genes showed an opposite direction of change in ND2.0 compared to other stock lines. One hypothesis to explain these findings is that the ND2.0 stock line might be inherently more heterogeneous than the others, increasing the risk that a gene-edited subline might differ from the broader parent ND2.0 population because the subcloning process had a greater impact than the gene edit. To address this possibility, single-cell RNA sequencing (scRNAseq) was compared for the ND2.0 and KOLF2.0 stock lines, and the population subject to cluster analyses (Figure 5A). Although both lines had gene expression profiles suggestive of pluripotent stem cells (Figure 5B), ND2.0 had subpopulations of cells not represented in KOLF2.0 (cluster 4, Figure 5A). Cluster 4 in the ND2.0 culture expressed high levels of genes typically associated with malignant transformation (Figure 5C and D). Comparison of Cluster 4 with cells in other clusters suggested biological pathways relating to DNA replication and repair, and cell division (Figure 5E).

In addition to identifying ND2.0 as being different from other stock lines, hierarchical clustering of samples using bulk gene expression (as heatmap) also revealed what appeared to be a batch effect for all stock lines (Supplementary Figure S2). Each stock line contributed three samples (A, B, C) collected by the same technician at different times less than one month apart under the same growing conditions. Despite identical culture conditions, one sample (A) from each showed a gene expression pattern distinct from the other two samples (B and C). Indeed, there were a considerable number of differentially expressed genes when comparing samples from batch A (across all four stock lines combined) against samples from batch B (*N* = 1856 genes) or C (*N* = 1043 genes). However, there were only four differentially expressed genes when comparing samples B and C. These results imply that batch B and C were virtually indistinguishable, but batch A was different.

## Discussion

The current studies provide empirical results from applying the gene-editing strategy for modeling LND, which is caused by mutations in the *HPRT1* gene. This disease is a useful model disease for several reasons. First, expression of *HPRT1* is markedly upregulated in rapidly proliferating cells, so mutations are likely to have direct relevance to the biology of stem cells.^55^ Second, LND is X-linked and recessive, so only one allele needs to be edited in iPSCs from males, with no possibility of epigenetic drift due to X-chromosome methylation during culture.^56^^,^^57^ Third, prior studies have demonstrated a strong genotype-phenotype relationship for this gene, such that pathogenic variants that eliminate enzyme activity consistently produce the same clinical and cellular phenotype.^45^ This observation means that mutations have a strong and consistent overall disease effect, even across individuals with different genetic backgrounds. Although the current study focused on LND, the methods applied and the results obtained are likely to be broadly relevant for many other genetic diseases.

The main goal was to assess reproducibility of gene editing across different stock lines and a secondary goal was to compare results to patient-derived lines. The study design intentionally incorporated the same *HPRT1* gene edit (so that results could not be attributed to different mutations), two different culture methods to prepare gene-edited cell lines (so that results could not be attributed only to technical methods), two different statistical strategies for detecting changes in gene expression (so that results could not be attributed only to the statistical analysis), and four different starting stock lines (so that the impact of the same gene edit could not be attributed solely to the cell line chosen). Three key findings deserve highlighting. First, making the same gene edit in different stock iPSC lines produces different results. Second, the gene-edited and case-derived strategies for modeling the same disease do not produce the same result. Third, unedited control stock lines and lines from healthy controls are not biologically equivalent. These findings are not likely to be limited only to the *HPRT1* gene and LND, but are more generally valuable for planning and interpreting other studies of gene-edited iPSCs for modeling genetic diseases.

### Making the same gene edit in different stock iPSC lines yields different results

An advantage claimed for the gene-editing strategy is that abnormalities are more readily detected because experimental variance associated with comparing iPSC lines across different genetic backgrounds is reduced by comparing a single mutation with an “isogenic” control. The most common strategy is to begin with a single stock line and study multiple gene-edited sublines as independent samples.^20^ The current studies reveal that results for gene editing vary according to starting stock line (Figure 2). Therefore, findings from a single stock line may be relevant only for the stock line chosen. The results may not be broadly relevant to the same gene edit in other stock lines. If this is the case, then, concerns related to the impact of genetic background for case-derived lines for disease modeling should be applied to gene-edited lines, and gene-edits should be made in multiple independent stock lines.

The extent of differences for the same gene edits across four different lines was unexpected. It seems likely that these differences result not only from the interaction of the gene edit with the different genetic backgrounds of the stock lines chosen, but additional technical issues, such as donor age, starting type of donor cell, method for introducing the gene edit, and selection of edited sublines. Other potential factors include pluripotency state and passage number, which are not usually monitored for publicly available stock lines, but known to be impacted by genetic and epigenetic drift in long-term cultures.^58^ The impact of these variables on iPSC modeling has not been methodically explored, but the current studies pointed to an additional important source of variance, which involves the isolation of a single subline from a more heterogeneous starting population. A problem with uniformity of the starting stock line was immediately obvious with the NN5200 line, where the original material obtained from the cell bank passed quality-control measures for karyotyping, but the culture population harbored a small proportion of cells that had acquired a small duplication in the long arm of Chr 20 which is common in iPSCs grown for extended periods in culture.^59^^,^^60^ Because this genomic change confers a growth advantage in culture, all edited sublines were found to carry it, and all NN5200 lines had to be discarded. A related problem was suspected for the ND2.0 lines, even though the karyotype was normal. Here, the large number of apparent differences in the gene-edited sublines may reflect isolation of the best-growing sublines from a more heterogenous starting population.

Experimental controls for the subcloning process may be needed, but they are rarely included in gene-editing studies. One potential solution to the problem of heterogeneity in the starting culture is to start by subcloning a single normal subline from the starting stock line, to mitigate heterogeneity in the starting population. This subclone can then be used to make multiple independent gene-edited lines and multiple independent non-edited lines for comparisons. This strategy will mitigate heterogeneity in the starting culture. However, this strategy is technically demanding and increases the risk of acquired mutations or epigenetic alterations that adapt the subline for optimal growth in culture. These adaptations may impact findings of gene editing unrelated to the disease, as shown next.

### Case-derived and gene-edited strategies generate different results for the same disease

The ideal scenario for disease modeling is that the case-derived and gene-editing strategies produce the same result. However, the robustness of any disease effect is likely to be an important factor. For both case-derived and gene-edited iPSCs, most of the *HPRT1* mutations studied (including the c.508C>T gene edit) are subject to nonsense-mediated decay with low mRNA levels (Tables 1 and 2). As a result, reduced expression of *HPRT1* mRNA was a robust and consistent finding. There was also a robust and consistent loss of HGprt enzyme activity across the case-derived and gene-edited lines (Tables 1 and 2), because the mutations studied all have a strong loss-of-function effect on the enzyme.^45^ Reduced *HPRT1* mRNA with loss of enzyme activity are all reliably detected because they are very robust effects of the disease. The effect of *HPRT1* mutations on the expression of other genes seems to be weaker, so results for differential gene expression from different modeling strategies and analytical approaches vary more.

One potential explanation for the different results obtained for case-derived versus gene-edited iPSCs is that the gene-edited lines all had the same c.508C>T mutation while the LND cases all had different mutations.^19^ This explanation seems unlikely, because the consequences of *HPRT1* mutations are determined by the overall impact of the mutation on HGprt enzyme function, not by different mutations.^45–47^ Regardless of the nature of the mutation, all null mutations produce null enzyme activity, similar downstream metabolic consequences, and a relatively stereotypical clinical phenotype. For both the gene-edited (Table 2) and case-derived lines,^19^ all mutations resulted in null enzyme activity, making it unlikely that differences in gene expression can be explained by different starting mutations.

Another potential explanation for the different results obtained for case-derived versus gene-edited iPSC lines is that the majority of differences in gene expression found by RNAseq are “noise” (false positives). This explanation seems unlikely, because introduction of known changes into the dataset produced an acceptably low rate of false positives when sample sizes were large. A better explanation relates to the different sources of variance in the case-derived versus gene-edited samples. This variance may come from experimental variables that often receive little attention in the literature such as donor characteristics (e.g. age, sex, genetic background), starting type of donor cell (e.g. fibroblast, leukocyte), method of derivation (e.g. retrovirus, Sendai virus, episome, mRNA), epigenetic memory, genetic and epigenetic drift in long-term culture, pluripotency state, and others.

When the effect of a disease is unknown, the best experimental design depends on whether the experimental goal is exploratory or hypothesis-testing. If the main aim is to test a preconceived hypothesis, then rigorous statistics to avoid incorrect conclusions from false positive findings is essential. Conversely, if the goal is exploratory and hypothesis-generating because the disease effects are not known, then it may be more important to avoid false negatives. It is worth noting that with commonly used parameters for gene expression changes, false positives may be more frequent than true positives, unless a relatively large number of lines are examined. For LND, the most consistent and unexpected findings involved three genes (*NR4A2*, *DNAJA1*, and *THAP9-AS1),* because abnormalities in these genes were found across different analytical strategies involving the case-derived iPSCs,^19^ the gene-edited stock lines, and in a prior study using a smaller number of case-derived lines.^44^ These genes are, therefore, probably disease-relevant and merit attention in future studies.

### Healthy control lines and normal stock lines are not equivalent

One important reason that gene-edited and case-derived iPSCs produce different results may also relate to how the baseline of “normal” gene expression is defined. A comparison between iPSC lines derived from healthy control individuals versus normal stock iPSC lines revealed unexpectedly large differences in gene expression. These differences are perhaps not surprising. Specific stock lines become popular because of robust growth in culture, so they likely have acquired adaptations for optimal growth in vitro that make them different from “average” control lines. It is widely known that cultured cells acquire genetic and epigenetic alterations over time that promote survival and growth.^10^^,^^59–63^ Indeed, biological pathways most different between these two types of control iPSC lines were related to DNA replication and cell growth.

This baseline difference in these two types of controls may impact the detection of any disease effect. For *HPRT1*, the associated enzyme plays an important role in recycling purines, which are essential building blocks for RNA and DNA. Accordingly, *HPRT1* is expressed at higher levels in rapidly dividing cells in comparison with post-mitotic cells.^64^ These observations predict that the impact of HGprt deficiency may vary among cells that may have adapted for rapid cell division, such as stock iPSC lines. An important implication of these observations is that the common strategy of comparing locally prepared patient-derived lines with normal stock lines obtained from a public bank should probably be avoided.

Several recent reports have suggested the need for a common reference line for all gene editing.^20^ The KOLF2.1J line has been recommended as universal reference line for gene editing,^20^ but recent studies have revealed small genomic deletions that may impact results.^20^ No cell line can retain complete genomic stability in culture. Duration in culture is known to lead to acquired mutations and genetic or epigenetic drift, and genomic integrity is usually assessed using the karyotype, micro-array platforms such as the Karyostat, or arrays targeting certain single nucleotide polymorphisms. None of these methods detects all potential mutations, and none detects epigenetic changes that affect cell growth in culture. Therefore, KOLF2.1J lines maintained in different laboratories are bound to diverge over time. The current studies show that the same gene edit produces different results in different starting stock lines, questioning whether a single iPSC line can ever serve as a universal “reference” line for all disease modeling. While use of the same stock line may address the problem of reproducibility of gene editing findings, this precision comes at the cost of disease relevance.

### Conclusions and recommendations

Many prior articles involving disease modeling imply that the gene-editing strategy is superior to the case-derived strategy. In fact, some have referred to the gene-editing strategy as the “gold standard” for disease modeling. The main arguments relate to mitigation of individual differences found among iPSC lines from different individuals, and reduction of variance by studying “isogenic” cells. For disease modeling with case-derived lines, prior studies have recommended including at least four unrelated cases and only 1 line per case, with statistical corrections when there are multiple sublines from the same case.^11^^,^^16^ For disease modeling with gene-edited lines, these recommendations are not usually applied. Instead, the more common approach is to study multiple sublines from a single stock line. Results from the current studies question this approach, by showing that different results come from editing different starting stock lines. If results vary according to starting stock line, then the same recommendation applied to case-derived iPSCs to study multiple independent sources should also be applied. The current results suggest the following recommendations:

1. The gene-editing strategy should not be considered a “gold standard” over the case-derived strategy for disease modeling using iPSCs; each strategy has different strengths and limitations.
2. In reference to the gene-editing strategy, the term “isogenic” should probably be discouraged unless whole genome sequencing is conducted, because unknown genetic or epigenetic changes unrelated to the targeted edit are likely to result from gene editing itself, genetic drift in culture, and the subcloning process itself. The term “gene-edited” is more accurate.
3. For disease modeling with gene-edited lines, comparisons may require more than one starting stock line to determine if results are unique to the starting line, or if they are reproducible findings that may be more generally disease-relevant.
4. Stock lines with high passage levels obtained from cell banks may harbor genetic or epigenetic adaptations for long term successful growth *in vitro*, and they are probably not ideal comparators as controls for locally prepared case lines from disease subjects.

## Supplementary Material

szaf065_Supplementary_Data

## Data Availability

Data are available by request from the corresponding author.
