# Supplementary material for: Modeling rare genetic disease with gene-edited induced pluripotent stem cells: relevance of the starting stock line
Source: Stem Cells Transl Med. 2025 Dec 16;14(12):szaf065. doi: 10.1093/stcltm/szaf065 (PMC12706867; doi:10.1093/stcltm/szaf065)

**Supplementary Information**

**Modeling Rare Genetic Disease with Gene-Edited Induced Pluripotent Stem Cells:**

**Relevance of the Starting Stock Line**

Ashok R. Dinasarapu, PhD ^1,‡^, Diane J. Sutcliffe, MPH ^1, ‡^, Erkin Ozel, MD ^1,2^, Anike Thite,BS ^1^, Lauren Grychowski, BS ^1,8^, Jasper E. Visser, MD, PhD ^3,4,5^, Ellen J. Hess, PhD ^6^, Sharon M. Kolk, PhD ^3^, and H.A. Jinnah, MD, PhD ^1,2,7,*^

1. Department of Neurology, Emory University School of Medicine, Atlanta, GA 30322 USA
2. Department of Pediatrics, Emory University School of Medicine, Atlanta, GA 30322 USA
3. Neurobiology Section, Donders Center for Neuroscience, Radboud University Nijmegen, The Netherlands
4. Department of Neurology, Radboud University Medical Center Nijmegen, The Netherlands
5. Department of Neurology, Amphia Hospital, Breda, The Netherlands
6. Department of Pharmacology, Emory University School of Medicine, Atlanta GA 30322 USA
7. Department of Human Genetics, Emory University School of Medicine, Atlanta, GA 30322 USA

**^*^Corresponding author**: H. A. Jinnah, M.D., Ph.D, Emory University School of Medicine, 101 Woodruff Circle, Atlanta, GA 30322 USA

hjinnah@emory.edu

Phone: 404-727-9107

^‡^These authors contributed equally to this study.

^8^Present address: Department of Neurology, Yale University School of Medicine, New Haven, CT, 06510, United States

**Running title**: Modeling Lesch-Nyhan Disease with Gene-Edited IPSCs

Figure S1. Confirmation of *HPRT1* gene editing. In addition to the karyotype and other quality checks, the RNAseq data from the final cell samples were interrogated to assess consequences of gene editing at the intended target region. The entire *HPRT1* sequence was evaluated, but only the relevant site is shown. The *HPRT1* sequences from all 3 samples of each of the 4 stock lines had a normal sequence, including a “C” at position 508. The *HPRT1* sequences from all 3 gene-edited lines from all 4 stock lines confirmed a C>T substitution at position 508. One of the edited lines from one of the stocks (NCRM1.C9E4) also appeared to have deletion of a single “G”4 bases away from the target at position 512. However, this additional mutation is not likely to be relevant because it comes after the newly introduced stop codon.

Figure S2. The heatmap was generated using the ComplexHeatmap R package (v2.20.0), available at https://github.com/jokergoo/ComplexHeatmap. Hierarchical clustering was performed using Euclidean distance metric and complete-linkage clustering. The scaled gene expression levels were colored according to the Z-score, with yellow color indicating lower expression values (Z-score < 0), purple indicating higher expression values (Z-score > 0) and green represents mean expression value (Z-score = 0).


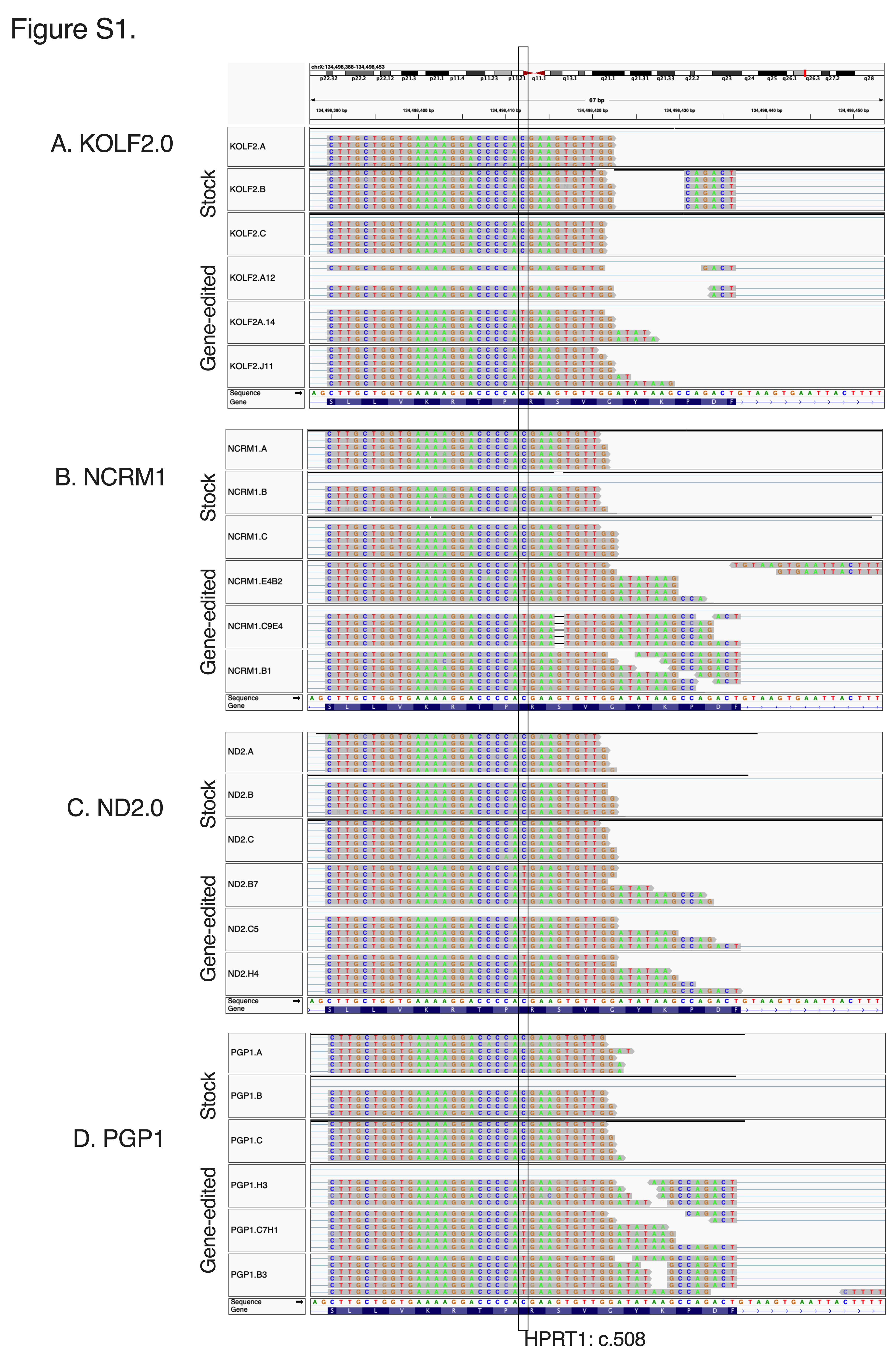


Figure S2.


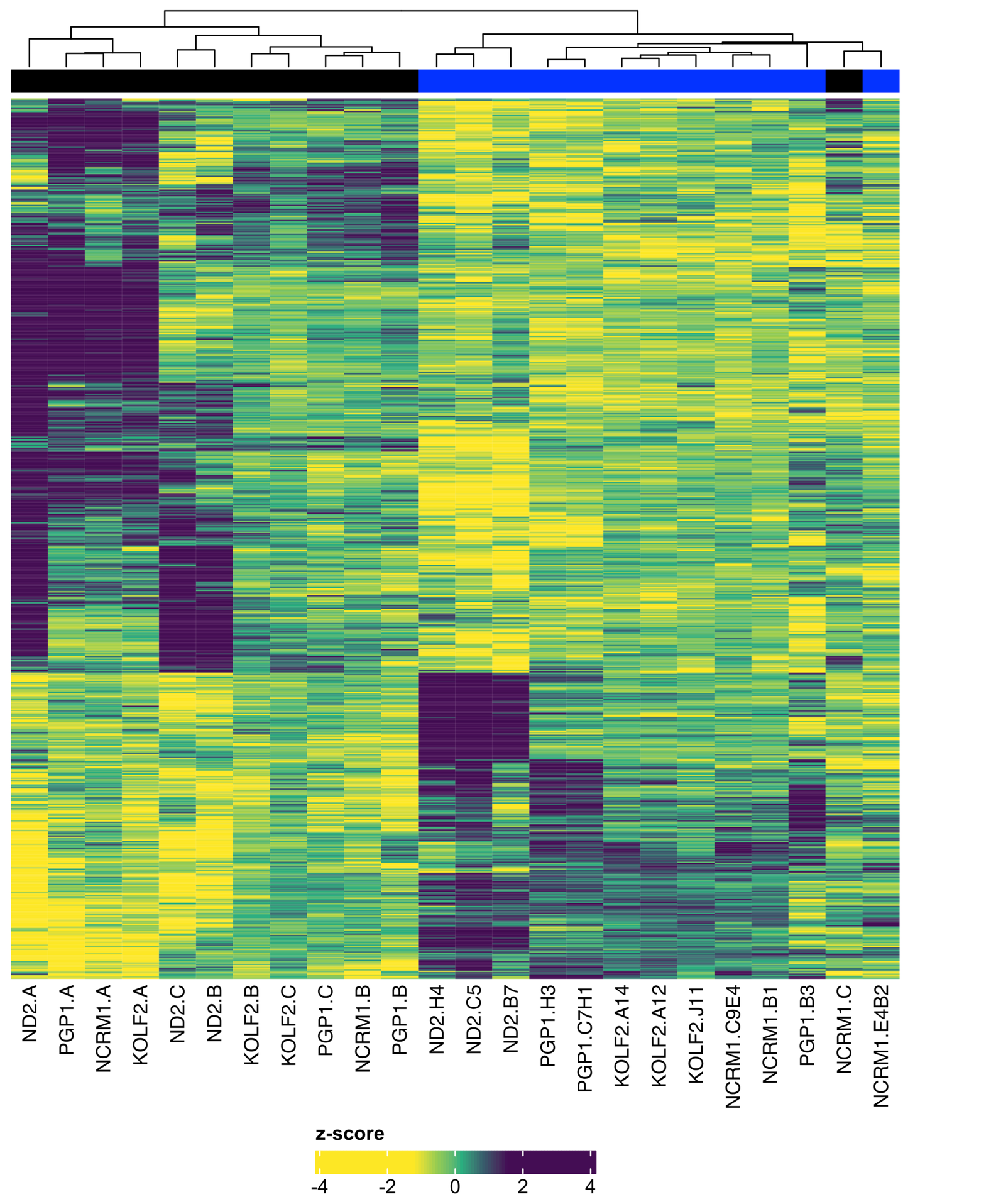

Supplement: szaf065_Supplementary_Data [file szaf065_supplementary_data.zip › 20-Nov-2025_053916_Supplementary_Figures_1-2.docx]
